# 

**Published:** 1885-04

**Authors:** 


					﻿WWole Number, 304.
APRIL, 1885.
Vol. L., Number 4.
TELE
CHICAGO MEDICAL JOURNAL AND EXAMINER
EDITORS:
S. J. JONES, M.D., LL.D.
W. W. JAGGARD, A.M , M.D.
HAROLD N. MOYER, M.D.
CHICAGO:
PUBLISHED BY THE CHICAGO MEDICAL PRESS ASSOCIATION,
240 Wabash Avenue.
CLARK &. LONGLEY, PRINTERS, 163 AND 165 DEARBORN STREET, CHICAGO.
				

